# Partially covered or uncovered metal stent efficacy in malignant unresectable distal biliary obstruction (METARSI): Randomized multicenter trial

**DOI:** 10.1055/a-2795-7703

**Published:** 2026-02-17

**Authors:** Silvia Cocca, Flavia Pigò, Helga Bertani, Roberta Rea, Giuseppina Pontillo, Michele Campigotto, Giuseppe Grande, Salvatore Russo, Margherita Marocchi, Marinella Lupo, Gian Maria Prati, Giovanni Aragona, Raffaele Manta, Carmelo Barbera, Fabio Monica, Francesco Di Matteo, Rita Conigliaro, Santi Mangiafico

**Affiliations:** 1 Gastroenterology and Endoscopy Unit, Azienda Ospedaliero Universitaria Policlinico di Modena, Modena, Italy; 2 220431 Operative Digestive Endoscopy, Campus Bio-Medico University Hospital, Rome, Italy; 3 UOC Endoscopia Digestiva, AOU S. Giovanni di Dio e Ruggi d'Aragona, plesso "Gaetano Fucito", Mercato San Severino (SA), Salerno, Italy; 4 18703 Gastroenterologia ed Endoscopia Digestiva, Ospedale Santa Maria di Ca Foncello, Treviso, Italy; 5 18638 Gastroenterology and Hepatology Unit, Guglielmo da Saliceto Hospital, Piacenza, Italy; 6 Gastroenterology and Digestive Endoscopy Unit, ASL Toscana Nord-Ovest, Perugia, Italy; 7 Gastroenterology and Digestive Endoscopy Unit, USL Teramo, Teramo, Italy; 8 Gastroenterology and Endoscopy Unit, Ospedali di Trieste, Trieste, Italy; 9 220431 Operative Digestive Endoscopy Department, Campus Bio-Medico University Hospital, Rome, Italy; 10 220340 Digestive Endoscopy Unit, Azienda Ospedaliero-Universitaria di Modena Ospedale Civile di Baggiovara, Modena, Italy; 11 Gastroenterology and Endoscopy Unit, Azienda Ospedaliero Universitaria Policlinico “G. Rodolico-San Marco”, Catania, Italy

**Keywords:** Pancreatobiliary (ERCP/PTCD), Strictures, ERC topics

## Abstract

**Background and study aims:**

There is no consensus regarding optimal selection of self-expandable stents (SEMS) for biliary drainage with endoscopic retrograde cholangiopancreatography (ERCP). This study compared stent dysfunction, patient survival, and adverse events in patients with unresectable malignant distal biliary strictures (DBS) treated with ERCP, randomized to partially covered (PC-SEMS) or uncovered (U-SEMS) stents

**Patients and methods:**

A prospective, multicenter, randomized controlled trial was performed in adult patients with DBS (March 2021-February 2023) with 12-month follow-up. Analyses were conducted according to intention-to-treat (ITT) and per-protocol (PP) principles. Procedural and post-procedural outcomes were evaluated using PP analyses and stent patency was evaluated with Kaplan-Meier analysis.

**Results:**

Among 261 patients, 130 were randomized to PC-SEMS and 131 to U-SEMS. Baseline features were similar between groups (mean age 73 ± 11 years; 51% male). Most strictures were due to pancreatic adenocarcinoma (75%), and 49% of patients had metastatic disease. Overall, stent dysfunction was comparable (11% vs 14%; *P* = 0.70). No significant differences were observed in patient survival (108 vs 100 days). A trend toward higher procedure-related complications was noted with PC-SEMS (2% vs 7%; not significant).

**Conclusions:**

PC-SEMS and U-SEMS in unresectable DBS showed comparable patency, survival, and stent dysfunction rates, with tumor ingrowth rarely observed and a trend toward more procedure-related complications in PC-SEMS. In this group with limited survival, there was no observed patency advantage with PC-SEMS. Potential benefit of PC-SEMS in populations with longer prognosis warrants further study.

## Introduction

It is estimated that 80% to 95% of biliary strictures in adults are malignant 1 2 . The most common cause of malignant distal biliary duct stricture (DBS) is pancreatic adenocarcinoma (extrahepatic). Other causes include cholangiocarcinoma, ampullary carcinoma, lymphoproliferative disorders, metastatic disease, and other local cancers (gallbladder and liver malignancies) 3 . Common clinical symptoms of malignant DBS are painless jaundice due to obstruction of bile flow, which can lead to various adverse events (AEs), including ascending cholangitis, vitamin and fat malabsorption, and coagulopathy complications 2 4 . Typically, patients with DBS have poor quality of life and prognosis, with an overall 5-year survival rate of less than 5%. It has been reported that at time of diagnosis, most lesions (70%) are unfit for curative tumor surgery, due to late symptom presentation and typical onset in elderly patients unfit for surgery. Therefore, palliative strategies are most commonly adopted 1 3 5 .

European guidelines recommend biliary drainage for biliary decompression for selected cases involving cholangitis, severe symptomatic jaundice, delayed demolitive tumor surgery, or neoadjuvant chemotherapy in jaundiced patients 4 . Drainage can be performed with various interventional strategies, including endoscopic, radiologic, or surgical approaches 4 6 .

The European Society of Gastrointestinal Endoscopy (ESGE) recommends endoscopic retrograde cholangiopancreatography (ERCP) because it is less invasive with a better cost-benefit ratio than surgery or percutaneous interventions 4 6 7 and has a lower risk of tumor seeding and complications compared with radiologic approaches 3 4 . If ERCP fails, ESGE recommends an endoscopic ultrasound (EUS)-guided approach 4 5 .

ERCP in patients with DBS can be performed with deployment of plastic or metallic self-expandable stents (SEMS). Compared with plastic, SEMS have been associated with lower risk of stent dysfunction and cholangitis and fewer reinterventions 6 8 . Commercially available SEMS are fully covered (FC-SEMS), uncovered (U-SEMS), or partially covered (PC-SEMS).

FC-SEMS were developed to prevent stent occlusion by tumor/tissue ingrowth with introduction of an internal covered mesh. However, although FC-SEMS are less likely to permit tumor ingrowth, no clear patency advantages associated with FC-SEMS have been reported. High rates of tumor overgrowth around the stent and sludge formation, coupled with risk of migration, have been observed 9 10 . Theoretical risk of cholecystitis in the case of coverage of the cystic duct by stent obstruction has also been highlighted. Therefore, PC-SEMS with uncovered flare ends were introduced. Both U-SEMS and PC-SEMS are permanently deployed and are most frequently selected for patients with DBS who are unfit for curative tumor surgery 11 12 .

A consensus regarding optimal stent selection between PC-SEMS and U-SEMS in ERCP for DBS is lacking. Several studies have compared FC-SEMS and U-SEMS; randomized controlled trials (RCTs) were performed in Canada and South Korea and authors concluded that PC-SEMS do not seem to provide benefits compared with U-SEMS 13 14 15 . Park et al. called for more RCTs to clarify the optimal role of PC-SEMS. Our study provides evidence for a European population. Further, our data contribute to the number of DBS patients enrolled in RCTs for future meta-analyses or to provide further evidence for development of a consensus.

The primary aim of this study was to compare overall stent dysfunction in DBS with PC-SEMS and U-SEMS. Secondary aims were to evaluate technical and clinical success, recurrent biliary obstruction/stent patency time, patient survival, and incidence of procedural-related complications.

## Patients and methods

### Study design

This investigator-led, prospective, multicenter RCT was conducted between March 2021 and December 2022 in four Italian centers (Azienda Ospedaliero-Universitaria of Modena, Campus BioMedico University of Rome, Cattinara University Hospital of Trieste and Hospital of Piacenza-Piacenza). The study was approved by the Ethics Committee of the coordinating center (Comitato Etico Area Vasta Emilia Nord, prot n.947/2020) and participating centers. The study was registered on ClinicalTrials.gov, protocol number: NCT04431427.

### Study population

Consecutive subjects undergoing ERCP for placement of SEMS for a malignant DBS during the study period were evaluated for study enrolment. Criteria for study inclusion specified: 1) patient age > 18 years; 2) neoplasia of the biliary or pancreatic tract unsuitable for surgical resection at the time of the ERCP procedure; 3) malignant distal biliary obstruction (≥ 1 cm distal to the biliary hilum); 4) assessment with the Karnofsky performance score; and 5) informed consent for study participation. Exclusion criteria specified: 1) history of biliary tract surgery (other than cholecystectomy); 2) previous biliary SEMS placement; and 3) coagulopathy (INR > 1.5 or platelet count < 50,000).

Karnofsky performance score, widely used in oncology and palliative care, evaluates disease burden and treatment efficacy on a scale ranging from 0 (death) to 100 (normal functioning). Scores were classified as mild (80–100), moderate (50–70), and severe impairment (< 50) 16 .

Comorbidities, diagnoses of malignancy, and Karnofsky performance scores were based on clinical, laboratory, radiologic, and/or pathologic findings. Disease stages and neoadjuvant chemotherapy and/or radiotherapy eligibility were determined from computed tomography and EUS findings. Data were collected at each participating center in dedicated case report forms and Excel databases. A central data manager combined the local databases into a single study dataset for analysis.

### Randomization

Patients were randomly allocated in a 1:1 ratio using a block size of four, stratified according to trial site. No competitive policy for the patient recruitment process was used. Patients were assigned to each group by a central registration method. After medical classification of study suitability, endoscopic procedure feasibility was determined when biliary duct access was achieved. The case was then communicated to a data manager at the coordinating center for electronic patient randomization to PC-SEMS or U-SEMS. Crossovers were permitted in cases in which multidisciplinary consideration for patient future surgery changed during the ERCP procedure.

### Procedures

All patients underwent ERCP in a supine or lateral position using a standard duodenoscope under deep sedation. Prophylaxis of post-ERCP pancreatitis (indomethacin or diclofenac suppository and Ringer's Lactate infusion) was performed in all patients without specific contraindications 17 . Access to the papilla was achieved directly. In cases of difficult biliary cannulation (defined as > 5 contacts with the papilla or > 5 minutes of cannulation attempts or > 1 pancreatic duct cannulation/opacification 17 ), a precut biliary sphincterotomy (fistulotomy [needle-knife sphincterotome], transpapillary septotomy [standard sphincterotome]), or endoscopic-radiologic rendezvous (salvage technique) was performed.

After biliary access, features of the stricture (such as angulation and length) were evaluated with cholangiography. Cystic duct occlusion (opacification during cholangiography) and stent placement covered the cystic duct origin were recorded. Stents were placed under fluoroscopic and endoscopic guidance by experienced pancreaticobiliary endoscopists. Stent length was chosen to extend approximately 1 cm beyond both the proximal and distal stricture margins. Available stents were WallFlex (Boston Scientific Japan, Tokyo, Japan), ComVi (Taewoong, South Korea) and Evolution (CookMedical, Livery, Ireland).

All patients received periprocedural antibiotic prophylaxis. In cases of fever with positive blood cultures, antibiotic treatment was prescribed for 7 days.

### Follow-up

Follow-up consisted of clinical visits or telephone contact at 3, 6, 9, and 12 months (± 3 weeks). Laboratory exams were requested between 3 to 6 weeks post-procedure and at each subsequent follow-up point.

Recurrent biliary obstruction was suspected in patients with clinical symptoms of obstructive jaundice or cholangitis and evaluated for reintervention via ERCP.

Patients unreachable by phone were considered lost to follow-up. Analyses were performed using intention-to-treat (ITT) and per-protocol (PP) approaches, with patients censored at time of the most recent follow-up.

Technical, procedure, and follow-up data for procedure-related complications and recurrent biliary obstruction were recorded.

### Definitions and endpoints

#### Primary endpoints

Overall stent dysfunction was defined as loss of adequate biliary drainage due to occlusion or migration requiring endoscopic reintervention. Time to stent patency was measured from initial SEMS placement to occlusion or migration *.*

#### Secondary endpoints

Technical success was defined as SEMS deployment in the intended location with sufficient coverage of the stricture 18 19 , technical radiological stent visualization, and flow of contrast medium or bile through the stent 18 20 .

Clinical success followed ESGE criteria 4 : bilirubin < 2 mg/dL within 3 weeks if baseline bilirubin < 10 mg/dL, or within 6 weeks if baseline bilirubin ≥ 10 mg/dL.

Suspected obstruction and migration were confirmed during ERCP reintervention.

Procedure-related complications included pancreatitis, cholecystitis (acute), cholangitis, stent malfunction (incomplete opening), bleeding/ulceration, and perforation, registered within 30 days 17 21 . Early complications were defined as ≤ 7 days; late complications > 7 days. Early stent dysfunction was defined as incomplete drainage due to sludge or incomplete expansion within ≤ 7 days. Early stent dysfunction was defined as incomplete drainage of the stent (from sludge or incomplete opening) ≤ 7 days. Recurrent biliary obstruction was defined as loss of adequate biliary drainage (due to occlusion or migration) requiring endoscopic reintervention > 7 days from base intervention. Adjusted patency time excluded early dysfunction cases (< 7 days).

Stent migration was defined as symptomatic dislodgement from the intended and original implant position, confirmed by imaging and/or clinical evidence of cholangitis or obstructive jaundice. Patient survival was defined from initial stent placement to all-cause mortality or lost to follow-up.

### Statistics

Previous pooled results reported an overall stent dysfunction relative risk of 0.85 (absence of recurrent symptomatic biliary obstruction was equivalent to the duration from primary stent placement to stent dysfunction), corresponding to an incidence of 25% in U-SEMS patients 22 . Assuming a 15% decrease in stent dysfunction with PC-SEMS, with an alpha-error of 0.05 and a power of 0.8, calculations approximated 100 patients were required for each group. Accounting for drop-outs and competing risks (surgical resection and high mortality within 1-year) target enrollment was increased by 30%.

Continuous variables were expressed as mean ± standard deviation or median and interquartile range (IQR) according to parametric or non-parametric distribution. Categorical variables were expressed as percentage. Patency was evaluated with the Kaplan-Meier method and compared with the log-rank test. Unadjusted and adjusted patency times were calculated. Patients with patent stents were censored at the last follow-up or death; surviving patients were censored at their last follow-up.

Group comparisons used chi-square or Fisher’s exact test for categorical variables and *t* -test or Wilcoxon test for continuous variables, according to distribution. ITT and PP analyses were performed. Protocol deviations (such as randomization before biliary cannulation) and untreated patients were excluded from PP analysis. *P* <.05 was considered significant. The analysis was conducted with the Stata statistical software, version 13.1 (StataCorp, Texas, United States).

## Results

### Patient characteristics

**Fig. 1:**
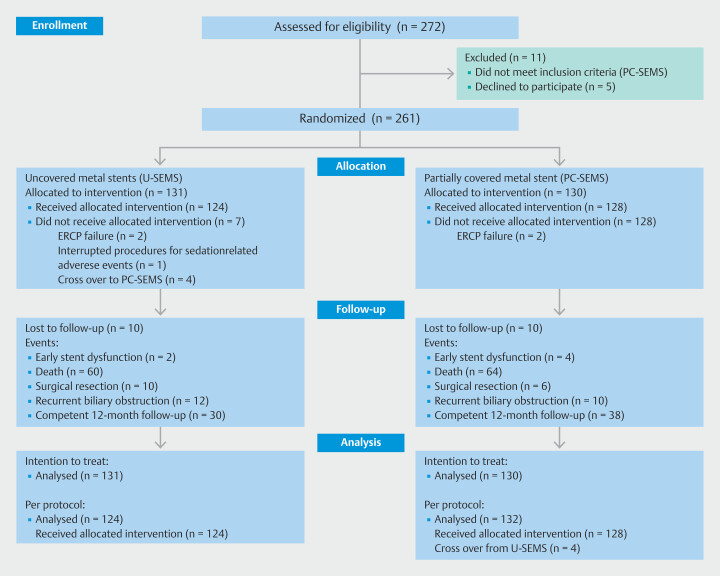
Consort flow diagram.

Of the 272 patients assessed for the present study, 261 met inclusion criteria and were enrolled. Of these, 131 were randomized to U-SEMS and 130 to PC-SEMS. Allocated interventions were not performed in two and seven patients, respectively. In both arms, 10 patients were lost to follow-up ( Fig. 1 ) 23 .

Patients included in ITT and PP analyses are shown in Table 1 . No statistically significant differences were observed between treatment groups at baseline with either analysis. Mean Karnofsky performance scores were mild. Mean age was 73 ± 11 years and 51% were male in the total population. The most commonly reported comorbidities were cardiac (30%) and metabolic diseases (18%) and hypertension (25%). Most DBS cases were due to pancreatic adenocarcinoma (75%). Patients had either locally advanced neoplasia (51%) or metastatic disease (49%) and just over half were classified as candidates for neoadjuvant chemotherapy or radiotherapy (54%).

**Table:** **Table 1** Baseline patient characteristics: Intention-to-treat and per-protocol analyses.

	**Intention to Treat**				**Per Protocol**			
	**All patients** **(n = 261)**	**U-SEMS (n = 130)**	**PC-SEMS (n = 131)**	***P* value **	**All patients (n = 256)**	**U-SEMS (n = 124)**	**PC-SEMS (n = 132)**	***P* value **
Characteristics								
Age (years), mean ± SD	73.5 ± 10.5	74.1 ± 9.8	73.9 ± 11.7	0.61	73 ± 11	74.5 ± 10.7	73.4 ± 12.8	0.59
Sex (male), number (%;95% CI)	132 (51%)	58 (44.6%)	74 (56;48–65)	0.06	130 (51%)	60 (48;39–58)	70 (53;44–62)	0.72
Comorbidities, number								
Hypertension	66 (25%)	28 (21;15–29)	38 (29;22–37)	0.20	64 (25%)	30 (24;17–33)	34 (26;19–34)	0.88
Cardiac disease	78 (30%)	32 (25;18–33)	46 (35;27–44)	0.07	74 (29%)	32 (25;18–34)	42 (32;24–40)	0.33
Metabolic disease	46 (18%)	18 (14;9–21)	28 (21;15–29)	0.14	44 (17%)	20 (16;10–24)	24 (18;12–26)	0.74
Respiratory disease	24 (9%)	12 (9;5–16)	12 (9;5–15)	10.0	22 (8%)	14 (11;6–18)	8 (6;3–12)	0.18
Neurological disease	24 (9%)	12 (9;5–16)	12 (9;5–15)	10.0	22 (8%)	14 (11;6–18)	8 (6;3–12)	0.18
Renal disease	20 (8%)	8 (6;3–12)	12 (9;5–15)	0.48	18 (7%)	10 (8;4–14)	8 (6;3–12)	0.62
Liver disease	16 (6%)	2 (1;0.1–6)	14 (11;6–17)	10.0	12 (5%)	6 (5;2–12)	6 (4;2–10)	10.0
Gastrointestinal disease	10 (4%)	2 (1;0.1–6)	8 (6;3–12)	0.10	6 (2%)	2 (2;0.1–6)	4 (3;1–8)	0.68
Other	10 (4%)	2 (1;0.1–6)	8 (6;3–12)	0.10	10 (4%)	2 (2;0.1–6)	8 (6;3–12)	0.10
No. comorbidities, number (%;95% CI)								
None	79 (30%)	36 (27;21–36)	43 (33;25–41)	0.41	76 (30%)	32 (25;18–34)	44 (33;25–42)	0.21
1	100 (38%)	54 (35;33–50)	46 (35;27–44)	0.30	102 (40%)	56 (45;36–54)	46 (35;27–44)	0.09
(≥ 2)	82 (31%)	40 (31;23–39)	42 (32;25–40)	0.89	78 (30%)	36 (29;21–38)	42 (32;24–40)	0.51
Etiology of distal malignant biliary obstruction, number (%; 95% CI)								
Pancreatic adenocarcinoma	196 (75%)	96 (74;66–81)	100 (76;68–82)	0.66	188 (73%)	92 (74;66–82)	96 (73;64–80)	1
Distal cholangiocarcinoma	20 (8%)	12 (9;5–16)	8 (6;3–12)	0.36	20 (8%)	12 (10;5–16)	8 (6;3–12)	0.52
Ampullary adenomas	25 (19%)	10 (8;4–14)	15 (11;7–18)	0.40	28 (11%)	8 (6;3–12)	20 (15;10–22)	0.15
Metastatic disease	20 (8%)	12 (9;5–16)	8 (6;3–12)	0.36	20 (8%)	12 (10;5–16)	8 (6;3–12)	0.52
Disease stage, number (%; 95% CI)				0.90				0.80
Locally advanced neoplasia	134 (51%)	66 (51;42–59)	68 (52;43–60)		134 (53%)	66 (53;44–62)	68 (51;43–60)	
Metastatic neoplasia	127 (49%)	64 (49;41–58)	63 (48;40–57)		122 (48%)	58 (47;38–56)	64 (48;40–57)	
Gallbladder lithiasis, number (%; 95% CI)	16 (6%)	6 (5;2–10)	10 (8;4–14)	0.44	14 (5%)	8 (6;3–12)	6 (4;2–10)	0.71
History of cholecystectomy, number (%; 95% CI)								
Total bilirubin (mg/dL)	11.2 ± 7.6	12.4 ±7.3	11.2 ± 8.3	0.30	11.8± 7.7	12.5 ± 7.2	11.0 ± 8.1	0.28
Direct bilirubin (mg/dL)	7.8 ± 5.2	8.2 ± 4.8	7.4 ± 5.5	0.51	7.7 ± 5.1	8.0 ± 4.9	7.3 ± 5.4	0.49
Alanine aminotransferase (IU/L)	198.1 ± 236.6	196.7 ± 189.2	212.6 ± 291.5	0.37	202.0 ± 245.8	192.8 ± 199.2	212.6 ± 291.5	0.66
Gamma-glutamyltransferase (IU/L)	689.1 ± 568.1	757.5 ± 610.3	614.6 ± 514.0	0.43	679.0 ± 571.2	754.2 ± 620.1	604.5 ± 523.1	0.34
Alkaline phosphatase (IU/L)	556.1±463.0	597.7±486.1	512.1±440.2	0.38	556.8 ± 463.9	597.7 ± 486.1	512.1 ± 440.2	0.38
Neoadjuvant chemotherapy or radiotherapy candidates, number (%;95% CI)	140 (54%)	66 (51;42–59)	74 (56;48–65)	0.38	138 (54%)	68 (55;46–64)	70 (53;44–62)	0.86
Karnofsky performance scores, mean ± SD	82.5 ± 10.2	81.3 ± 11.0	82.4 ± 10.9	0.64	81.3 ±10.9	80.9 ± 10.4	81.7 ± 11.3	0.94
CI, confidence interval; SD, standard deviation.								

In PP analysis, direct bile duct access was achieved in over 80% of patients, with a significantly higher representation in the U-SEMS group ( *P* = 0.04). In cases of ERCP failure, a rendezvous procedure was performed. SEMS length ranged from 40 to 80 mm (10-mm diameter). Intentional stent deployment over the origin of the cystic duct was performed in 25% of procedures. Procedure characteristics are summarized in Table 2 .

**Table:** **Table 2** ERCP procedure characteristics (per-protocol analysis).

**Characteristics**	**All patients (n = 256)**	**U-SEMS (n = 124)**	**PC-SEMS (n =132)**	***P* value **
Bile duct access, number (%; 95% CI)				
Direct	212 (83%)	112 (90; 84–95)	100 (76; 68–83)	0.04
Precut	36 (14%)	10 (8; 4–14)	26 (20; 13–28)	0.07
Trans-pancreatic septotomy	8 (3%)	2 (2; 1–6)	6 (4; 2–10)	0.61
Sphincterotomy, number (%; 95% CI)	194 (76%)	90 (72; 64–80)	104 (79; 71–85)	0.53
Cystic duct infiltration number (%; 95% CI)	38 (15%)	24 (19; 13–27)	14 (11; 6–17)	0.28
Size of study stent, length in mm × diameter in mm, number (%; 95% CI)				
40 × 10	68 (26%)	26 (21; 14–29)	42 (32; 24–40)	0.23
60 × 10	174 (68%)	88 (71; 62–79)	86 (65; 56–73)	0.57
80 × 10	14 (5%)	10 (8; 4–14)	4 (3; 1–8)	0.26
Stent placement over cystic duct origin, number (%;95% CI)	64 (25%)	32 (26; 18–34)	32 (24; 17–32)	0.84
Stent model, number (%; 95% CI)				
WallFlex	190 (74%)	88 (71; 62–79)	102 (77; 69–84)	
ComVi	44 (17%)	14 (11; 6–18)	30 (23; 16–31)	
Evolution	22 (8%)	22 (18; 11–26)	N/A	
CI, confidence interval; ERCP, endoscopic retrograde cholangiopancreatography; PC-SEMS, partially- covered self-expandable stent; U-SEMS, uncovered self-expandable stent.				

Post-ERCP characteristics were similar between groups. In PP analyses, technical success was achieved in 100% of procedures and clinical success was registered in 68% (U-SEMS) and 71% (PC-SEMS). A trend toward more overall procedure-related complications was observed in the PC-SEMS group ( *P* = 0.07).

Two patients developed moderate pancreatitis within 7 days of the ERCP procedure; both patients were successfully managed with medical treatment. Acute cholecystitis developed in two patients from the PC-SEMS group; both patients were subjected to an ERCP reintervention with PC-SEMS removal and substitution with U-SEMS. Five patients developed cholangitis: four patients (PC-SEMS) without stent occlusion were treated with antibiotic therapy alone and the remaining patient had stent occlusion by biliary sludge (U-SEMS) and was treated with an endoscopic reintervention to insert a coaxial biliary plastic stent. Stent malfunction was registered in three patients (1 U-SEMS and 2 PC-SEMS), each requiring endoscopic placement of a coaxial plastic stent. No cases of bleeding or perforation were reported. All complications occurred within 7 days of the procedure. All post-ERCP data are summarized in Table 3 .

**Fig. 2:**
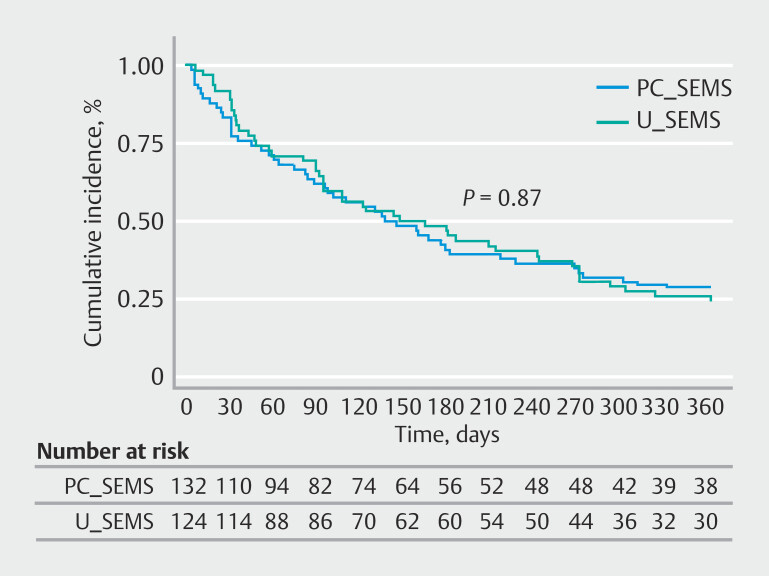
Patency rates.

Post-ERCP stent dysfunction was observed in 15 patients (11%; 95%CI 7–18) treated with U-SEMS and 17 patients (13%; 95% confidence interval [CI] 8–20) treated with PC-SEMS (not significant). Surgical resection rates were slightly higher in the U-SEMS group, which also had marginally longer follow-up duration. Recurrent biliary obstruction due to tumor ingrowth with or without biliary sludge impaction occurred in 22 (U-SEMS n = 10, PC-SEMS n = 10). Both cases of stent migration were observed in patients treated with PC-SEMS. All obstructions and migrations were treated with ERCP reinterventions with deployment of coaxial plastic stents. Patency rates were unstable (wide [CIs) and meaningful interpretation is difficult to make ( Fig. 2 ). Ten patients were lost to follow-up in each group; the overall rate was 7.7%. These patients did not attend scheduled visits and were unable to be reached by telephone.

**Table:** **Table 3** Post-procedure characteristics (intention to-treat and per protocol analysis).

	**Intention to-treat**			**Per Protocol Analysis**		
	**U-SEMS (n = 130)**	**PC-SEMS (n = 131)**	***P* value **	**U-SEMS (n =124)**	**PC-SEMS (n =132)**	***P* value **
Characteristic						
Technical success, number (%; 95% CI)	127 (98; 93–99)	129 (98; 95–100)	0.68	124 (100)	132 (100)	1.0
Clinical success, number (%; 95% CI)	86 (66; 57–74)	92 (70; 62–78)	0.50	84 (68; 59–76)	94 (71; 63–79)	0.58
Procedure related complications, number (%; 95% CI)						
Early adverse events	3 (3; 1–8)	8 (6; 3–12)	0.37	3 (2; 1–7)	9 (7; 3–13)	0.13
Pancreatitis	1 (1; 0.1–4)	1 (1; 0.1–4)	1.00			
Acute cholecystitis	0	2 (1; 0.1–5)	0.49			
Stent malfunction	1 (1; 0.1–4)	2 (1; 0.1–5)	1.00			
Bleeding	0	0	0			
Perforation	0	0	0			
Cholangitis (without stent occlusion)	1 (1; 0.1–4)	4 (3; 0.1–6)	0.37			
Late adverse events	0	0	1.00			
Overall stent dysfunction rate, number (%; 95% CI)	15 (11; 7–18)	17 (13; 8–20)	0.85	14 (11; 6–18)	18 (14; 8–21)	0.70
Recurrent biliary obstruction (stent dysfunction rate), number (%; 95% CI)	13 (10; 5–16)	13 (10; 5–16)	1.00	12 (10; 5–16)	14 (11; 6–17)	1.00
Migration	0	2 (2;0.1–5)	0.23			
Occlusion	10 (8;4–14)	12 (9;5–15)	0.82			
Time stent patency, days (median, 25th-75th percentiles)	210 (27–270)	92 (5–201)	0.52	268 (34–273)	87 (5–193)	0.45
Time to stent patency adjusted, days (median, 25th-75th percentiles)	132 (57–273)	127 (61–219)	0.78			
Post-ERCP surgical resection, number (%;95% CI)	8 (6; 3–12)	6 (4; 2–10)	0.71			
Time to surgical resection, days (mean ± SD)	95.8 ± 45.9	83.0 ± 61.5	0.06			
Death, number (%;95% CI)	62 (50; 41–59)	64 (48; 40–57)	0.44			
Survival time, days (median, 25th-75th percentiles)	106 (34–168)	103 (36–177)	0.30	108 (47–215)	100 (33–178)	0.14
Follow-up						
Total time, days (median, 25th-75th percentiles)	132 (48–273)	109 (45–206)	0.22			
Lost to follow-up, number (%;95% CI)	10 (8; 4–14)	10 (7; 4–13)	1.0			
Time to lost follow-up, days (median, 25th-75th percentiles)	62 (31–181)	31 (31–61)	0.40			
CI, confidence interval; ERCP, endoscopic retrograde cholangiopancreatography; PC-SEMS, partially-covered self-expandable stent; U-SEMS, uncovered self-expandable stent.						

There were no differences in survival time between stent types over the 360-day observation period (range 34–177 days). Post-procedure characteristics are outlined in Table 3 .

## Discussion

This prospective investigator-led, multicenter, randomized trial reports similar stent dysfunction, patient survival, and procedure-related complications in patients with unresectable malignant DBS treated with ERCP with either U-SEMS or PC-SEMS. A trend toward a higher complication rate was observed in the PC-SEMS group.

ERCP is the preferred palliative treatment for patients with DBS, compared with surgery or percutaneous approaches. Among available stents, SEMS have been shown to provide superior patency and a more favorable cost-effectiveness profile than plastic stents 24 . However, evidence remains inconsistent regarding optimal SEMS design for DBS.

Many SEMS types are commercially available, including PC-SEMS, FC-SEMS, and U-SEMS. U-SEMS typically demonstrate higher rates of tumor ingrowth (16%-46%) 6 24 , whereas FC-SEMS are associated with increased sludge deposition, tumor overgrowth, stent migration, cholecystitis, and pancreatitis 15 17 . Few studies have directly compared PC-SEMS with U-SEMS; however, most dedicated investigations concur that PC-SEMS are associated with lower rates of tumor ingrowth and AEs, while maintaining comparable migration rates and overall patient survival to U-SEMS 17 .

A systematic review and metanalysis published in 2013 identified eight RCTs and three prospective studies reporting SEMS deployment in all types of malignant obstructions of the digestive tract 25 . Only a limited number of these patients had undergone ERCP specifically for DBS (range 3–307). The authors reported substudy outcomes demonstrating comparable rates of stent patency, overall survival, and stent dysfunction-free survival to those observed in our study. Yang et al. similarly found no significant differences in overall AEs, including pancreatitis and cholecystitis, over a 12-month follow-up period. Accordingly, the review concluded that FC-/PC-SEMS and U-SEMS perform equivalently in these metrics 25 . A more recent RCT in patients with unresectable malignant DBS treated with PC-SEMS and U-SEMS (n = 258 patients) reported no clear advantages in stent patency or overall survival, but underscored lower tumor ingrowth rates with PC-SEMS 17 .

Despite the theoretical advantage conferred by the internal mesh of PC-SEMS, our study found no difference in tumor ingrowth between groups. Park et al. Previously reported reduced tumor ingrowth with PC-SEMS and suggested that chemotherapy may enhance stent patency by reducing disease burden. However, our findings did not confirm these associations, despite similar rates of chemotherapy exposure across cohorts.

In our study, both cases of stent migration observed were treated with PC-SEMS. Because these events were detected early, we hypothesize that migration may have occurred before the flared ends had adequately embedded into the biliary duct wall. A single-center, three-armed RCT in patients with unresectable DBS reported longer stent patency, fewer AEs (including cholangitis and cholecystitis), and lower migration rates with PC-SEMS 26 . Although our trial similarly demonstrates comparable patency and survival between PC- and U-SEMS, we observed a trend toward higher complication rates in the PC-SEMS group.

Most patients were classified with a mild Karnofsky score at baseline. Nevertheless, most deaths were registered within 4 months. Although mortality rates were similar between treatment groups, overall survival in our study was shorter than that reported in comparable cohorts. Yokota et al 11 reported median survival periods of 172 and 161 days for PC-SEMS and U-SEMS in a similar patient cohort (treated with Wallflex SEMS), whereas our cohort demonstrated notably shorter survival (108 and 100 days). This discrepancy may reflect overestimation of patient baseline functional status, raising the issue of applicability of patient classification with the Karnofsky score in this patient population for meaningful comparison.

In addition, the relatively low number of AEs observed in our study may be attributable to limited patient survival, which shortened the window for late complications to manifest.

Performance of SEMS may also be influenced by underlying disease. A recent review comparing FC-SEMS and U-SEMS reported that locally advanced pancreatic cancer was associated with greater cumulative patency than other cancer types, whereas no significant differences were observed in metastatic pancreatic cancer, likely due to shortened survival 27 . In our study, the small number of recorded events precluded meaningful assessment of disease-specific effects on patency and survival. Notably, none of the patients with pancreatic adenocarcinoma were alive at 12-month follow-up, underscoring the aggressive clinical trajectory of this disease.

This study is mainly limited by a lower-than-expected number of events, protocol deviations, and potential inclusion of patients with moderate to severe disease burden. The original sample size calculation assumed a stent dysfunction rate of approximately 25%. Although the study had sufficient power to detect large differences, it was underpowered to detect the small absolute difference observed between arms. Consequently, this trial cannot exclude either equivalence or a small benefit of one stent over another. The initial protocol specified patient classification as “fit” and “unfit”; however, the limited number of events, short patient survival, and relatively high rate of loss to follow-up restricted the ability to perform meaningful post-procedure comparisons. Nonetheless, the proportion of patients lost to follow-up was in line with other RCTs 15 .

The authors emphasize that SEMS outcomes are also influenced by radial and longitudinal forces exerted on the biliary duct, the specific anchoring mechanism, and morphology of the obstruction, which were not evaluated in this study. Meta-analyses and larger multicenter studies are required for complete risk factor analyses. Generalizability of our study results is limited to settings with experienced endoscopists. Further, more recent technological advancements in SEMS, such as drug-eluting, antireflux, and multi-hole- EMS 28 , were not included in our study and are expected to further improve outcomes. Although biliary drainage can improve the clinical condition of DBS patients, assessment of all aspects of well-being in this patient population should be included in future research 29 .

Although our findings did not demonstrate differences in overall stent patency, PC-SEMS may still play a role in selected patient subgroups. In particular, in patients with longer expected survival or who may convert from unfit to fit for surgery after effective chemotherapy, the partial covering could theoretically reduce late tumor ingrowth and facilitate reintervention. In our cohort, late tumor ingrowth was not observed, likely due to short patient survival, which limited the time window in which ingrowth typically becomes clinically relevant and may have masked any potential difference between stent types. Although this theoretical advantage of PC-SEMS could not emerge within the timeframe and clinical characteristics of our study, future trials stratified by survival expectancy and treatment response are warranted to determine whether PC-SEMS offer a meaningful benefit in patients with better prognoses.

## Conclusions

Results from our prospective multicenter RCT indicate that, in the clinical scenario of malignant unresectable distal biliary strictures, the choice between PC-SEMS and U-SEMS does not substantially affect stent patency or survival. A trend toward a higher incidence of procedure-related complications with PC-SEMS was observed, highlighting the need for careful patient selection and further targeted investigations.
